# Low-field NMR with multilayer Halbach magnet and NMR selective excitation

**DOI:** 10.1038/s41598-023-47689-2

**Published:** 2023-11-30

**Authors:** Ahmad Telfah, Ahmed Bahti, Katharina Kaufmann, Enno Ebel, Roland Hergenröder, Dieter Suter

**Affiliations:** 1 Leibniz-Institut für Analytische Wissenschaften-ISAS-e.V., 44139 Dortmund, Germany; 2 Nanotechnology Center, The University of Jordan, Amman, 11942 Jordan; 3 Department of Physics, University of Nebraska at Omaha, Omaha, NE 68182 USA; 4 Experimental Physics III, TU Dortmund University, 44227 Dortmund, Germany; 5 Fachhochschule Dortmund-University of Applied Sciences and Arts, 44139 Dortmund, Germany

**Keywords:** Analytical chemistry, Chemical physics, Techniques and instrumentation

## Abstract

This study introduces a low-field NMR spectrometer (LF-NMR) featuring a multilayer Halbach magnet supported by a combined mechanical and electrical shimming system. This setup offers improved field homogeneity and sensitivity compared to spectrometers relying on typical Halbach and dipole magnets. The multilayer Halbach magnet was designed and assembled using three nested cylindrical magnets, with an additional inner Halbach layer that can be rotated for mechanical shimming. The coils and shim-kernel of the electrical shimming system were constructed and coated with layers of zirconia, thermal epoxy, and silver-paste resin to facilitate passive heat dissipation and ensure mechanical and thermal stability. Furthermore, the 7-channel shim coils were divided into two parts connected in parallel, resulting in a reduction of joule heating temperatures from 96.2 to 32.6 °C. Without the shimming system, the Halbach magnet exhibits a field inhomogeneity of approximately 140 ppm over the sample volume. The probehead was designed to incorporate a solenoidal mini coil, integrated into a single planar board. This design choice aimed to enhance sensitivity, minimize ${B}_{1}$ inhomogeneity, and reduce impedance discrepancies, transmission loss, and signal reflections. Consequently, the resulting linewidth of water within a 3 mm length and 2.4 mm inner diameter sample volume was 4.5 Hz. To demonstrate the effectiveness of spectral editing in LF-NMR applications at 29.934 MHz, we selectively excited hydroxyl and/or methyl protons in neat acetic acid using optimal control pulses calculated through the Krotov algorithm.

## Introduction

Nuclear magnetic resonance (NMR) spectroscopy is a quantitative, highly reproducible, non-selective, and non-destructive method used for diagnosis, disease progression monitoring, and therapeutic interventions^1^. In contrast to standard (high-field) NMR spectroscopy, acquisition and operational costs of low-field NMR (LF-NMR)^2^ are substantially low. LF-NMR is employed to measure small molecules^3^, large systems such as lipids^4^, polymers, and rigid solids^5,6^. Additionally, innovative medical applications in detecting functionalized paramagnetic compounds binding to disease markers^7–9^ and conducting metabolomics analyses of body fluids like saliva, urine, and cerebrospinal fluid via NMR spectra^10^. However, sensitivity is one of the main challenges for LF-NMR, compounded by signal overlap due to the reduced frequency dispersion of the chemical shift range^11^, resulting in severe signal superposition in NMR spectra of complex mixtures, complicating identification and quantification.

Halbach magnets, based on Halbach arrays that arrange permanent magnets to generate strong, highly homogeneous magnetic fields^12–14^, have enabled the development of new-generation portable NMR spectrometers with magnetic field strengths exceeding 3 T and inhomogeneity of less than 100 ppm^13,15,16^. Magnetic field homogeneity is defined by the amplitude of magnetic field strength divided by the mean magnetic field at the sweet spot of the magnet. Typically, Halbach magnets exhibit spatial field homogeneity values in the range of 20–2000 ppm^17,18^, which are inadequate for high-resolution NMR spectroscopy. Therefore, we constructed a wide-bore Halbach magnet consisting of three concentric Halbach arrays, equipped with an extra layer of quadrupole ferromagnetic cubic elements to guide the magnetic field in a closed loop^19^. Additionally, the design includes quadrupole triangular focusers (Fig. 3a) to concentrate the resultant magnetic field at the sweet spot^20^.

**Figure 1:**
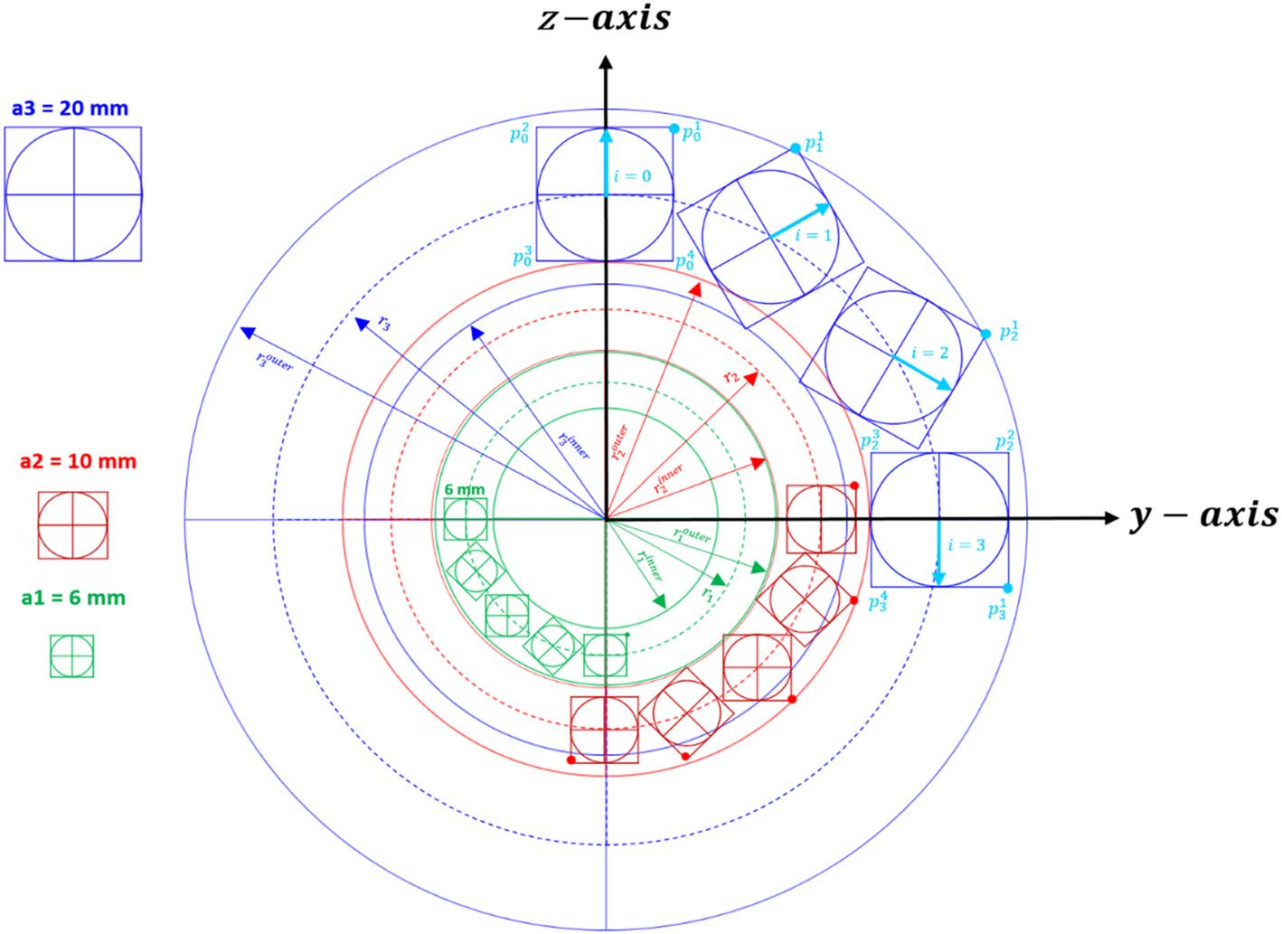
Construction principle of the Halbach cylinder, where identical magnets of size "a" are positioned with their center vectors, ${P}_{i}^{c}$ at angles ${\alpha }_{i}$ on a circle of radius $r$. The three-layered Halbach magnet was designed using AutoCAD, adhering to the Halbach condition for each individual layer to ensure constructive addition of the magnetic field (${B}_{z}$) while eliminating other directional magnetic components.

The potential of a rotatable Halbach layer as an integrated mechanical shim system was explored to complement the electrical shim system for achieving high-resolution NMR at low field strength. The mechanical shimming system consists of a concentric rotatable Halbach array (Fig. 5), providing a variable magnetic field amplitude in the z-y plane (${B}_{0}$ in the z-direction (Fig. 1)), thereby augmenting NMR magnetic field (${B}_{{z}_{0}}$)) homogeneity. Moreover, passive shimming was achieved by installing a ferromagnetic cylinder into the magnet bore, reducing the primary NMR magnetic field (${B}_{0}$) and allowing for magnetic field adjustments for lower NMR frequencies.

Optimal control theory was employed to design pulses suitable for specific NMR spectroscopy tasks, including selective excitation, inversion, decoupling, coherence transfer, and Hadamard encoding^21,22^. Pulse sequences were generated using the Krotov algorithm to guide spins to a desired final state in the presence of chemical shifts, RF inhomogeneities, and couplings. Innovatively, we designed optimal control pulses (OCP) for selective excitation to address low spectral dispersion^23^ enabling NMR spectroscopy at low magnetic fields. The LF-NMR spectrometer developed in this work can be further improved and customized for specific applications, including real-time and in-situ monitoring of target molecules and the suppression of interfering species. Additionally, it provides a contactless approach for identifying and quantifying target chemical compounds in multichemical mixtures using pre-calculated OCP sequences.

## Low-filed NMR multilayer Halbach magnet

A Halbach cylinder generates a stronger and more homogeneous magnetic field per unit mass compared to dipolar permanent magnets^24,25^. Furthermore, the generated magnetic field is oriented perpendicular to the cylinder's axis (Fig. 1), allowing for the incorporation of solenoidal r.f. coils and enabling NMR experiments where the magnetic field direction can be rotated relative to the sample^5,6^. A Halbach magnet is constructed by arranging identical cubic magnets in a circular array, resulting in constructive magnetic field addition within the ring's interior, leading to a dipolar field^26^. The concept of the NMR Halbach cylinder was expanded by implementing three nested Halbach arrays (Fig. 3) using 3D printing technology. Additionally, a rotatable shim cylinder is integrated to provide an adjustable compensating magnetic field for improved homogeneity^18^. As the z-axis does not align with the cylinder's symmetry, the magnetic field component of the rotational shim cylinder can contribute to the main magnetic field (${B}_{{z}_{0}}$) (Fig. 5). The cubic magnet elements ($i$) within the Halbach cylinder are positioned at an angle ${\alpha }_{i}=2\pi i/n$ (i = 0, 1,.., n−1), with their magnetization axis rotated relative to the z-axis (Fig. 1). Identical magnets with edge length ${a}_{l}$ (where l corresponds to the layer number, ranging from 1 to 3) are placed with their center vectors $({{}^{c}{\varvec{P}}}_{i})$, representing the vector from the center of the Halbach layer to the center of each magnet element (expressed in Eq. (1) and illustrated in Fig. 1). These magnets are positioned at an angle *α*_*i*_ on a circle of radius $r$. The Halbach cylinder's characteristics are defined by parameters such as the ring radius $r$ (indirectly related to magnet size), the number of cubic magnets ($n$), and the remanent magnetization^25,26^. The coordinates of each magnet element ${{\varvec{P}}}_{i}^{j}$, with *i* and *j* denoting the magnet and vertex identification, respectively, are described by a vector. These vectors are numbered clockwise from the center of each magnet and are defined as^13,27,28^.

1 $${{\varvec{P}}}_{i}^{j}= {{}^{c}{\varvec{P}}}_{i}+\frac{a}{\sqrt{2}} \cdot \left(\begin{array}{c}cos\left[\frac{2j-1}{4}\pi -\frac{4\pi }{n}\right]\\ sin\left[\frac{2j-1}{4}\pi -\frac{4\pi }{n}\right]\end{array}\right)\;\; \mathrm{and } \;\; {{}^{c}{\varvec{P}}}_{i}=r \cdot \left(\begin{array}{c}sin({\alpha }_{i})\\ cos({\alpha }_{i})\end{array}\right)$$

Where, $j$ ranges from $1$ to $4$ (representing the cube vertices), and $i$ varies from $0$ to $n-1$, where n corresponds to the number of magnets.

The inner (${r}_{inner}$), and outer (${r}_{outer}$) radii, measured from the center of each cube positioned at a radius (r) within a specific layer, are determined by Eq. (2):

2 $${r}_{inner}=r \cdot \left(1-\sqrt{2}\Xi \left(n\right)\right),\;\;{r}_{outer}=r \cdot (1+\sqrt{2}\Xi \left(n\right))$$

    Where, $n$ represents the number of magnetic elements, while r stands for the radius of the central cylinder line in each layer (*r*_*1*_: radius of layer 1, *r*_*2*_: radius of layer 2,* r*_*3*_: radius of layer 3). The parameter $\Xi (n)$ relates to the geometry and volume of the Halbach cylinder and can be approximated as $\Xi \left(n\right) \approx 2.26/(n-1.6)$ for $n > 4$^13,17^.

The vertex coordinates (${{\varvec{V}}}_{{\varvec{c}}}$) in polar coordinates are defined as:

3 $${{\varvec{V}}}_{{\varvec{c}}}={{}^{c}{\varvec{P}}}_{i}+ \sqrt{2} r\Xi \left(n\right)\cdot \left(\begin{array}{c}cos[\frac{2j-1}{4}\pi -\frac{4\pi }{n}i]\\ sin[\frac{2j-1}{4}\pi -\frac{4\pi }{n}i]\end{array}\right)$$

$j=1, .., 4$ describing the rotation of each magnet, $i=0, 1,\dots , n-1$ ($n$ represents the magnet numbering).

The magnetic moment (m) is calculated as $m=M\cdot V$, where $M$ and $V$ correspond to the magnetization and the volume of a single cube magnet, respectively. For cube magnets, the volume of a cylindrical Halbach array can be expressed as $2r\Xi \left(n\right)$, leading to $m=M{(2 r\Xi \left(n\right))}^{3}$.

In the case of the multilayer Halbach magnet, the z component of the magnetic field as a function of x is the summation of the contributions from the three layers:

4 $${B}_{z}\left(x\right)={B}_{R}\sum_{l=1}^{3}\frac{{\left(\frac{3}{\uppi } {n}_{l}\Xi \left({n}_{l}\right)\right)}^{3}}{{\left(1+{\left(\frac{x}{{r}_{l}}\right)}^{2}\right)}^{3/5}}$$

$l$ denotes the layer order, starting from the concentric point of the layers (${n}_{l}$: ${n}_{1}=16, {n}_{2}=16, {n}_{3}=12$) and ${B}_{R}={\mu }_{0}\cdot M$ represents the remanence of the individual magnets. To calculate the magnetic field of the multilayer Halbach magnet, which consists of multiple stacks in the x-direction, each Halbach stack (comprising 3 rings) can be treated as a dipole. The superposition of these terms leads to the following expression:

5 $${B}_{z}\left(x\right)=\frac{6{B}_{R}}{\uppi }\sum_{K=0}^{\infty }\sum_{l=1}^{3}\frac{{n}_{l}{((\Xi \left({n}_{l}\right))}^{3}}{{({1+{((\Xi \left({n}_{l}\right))}^{2}(1+2k)}^{2})}^{5/2}}$$

**Figure 2:**
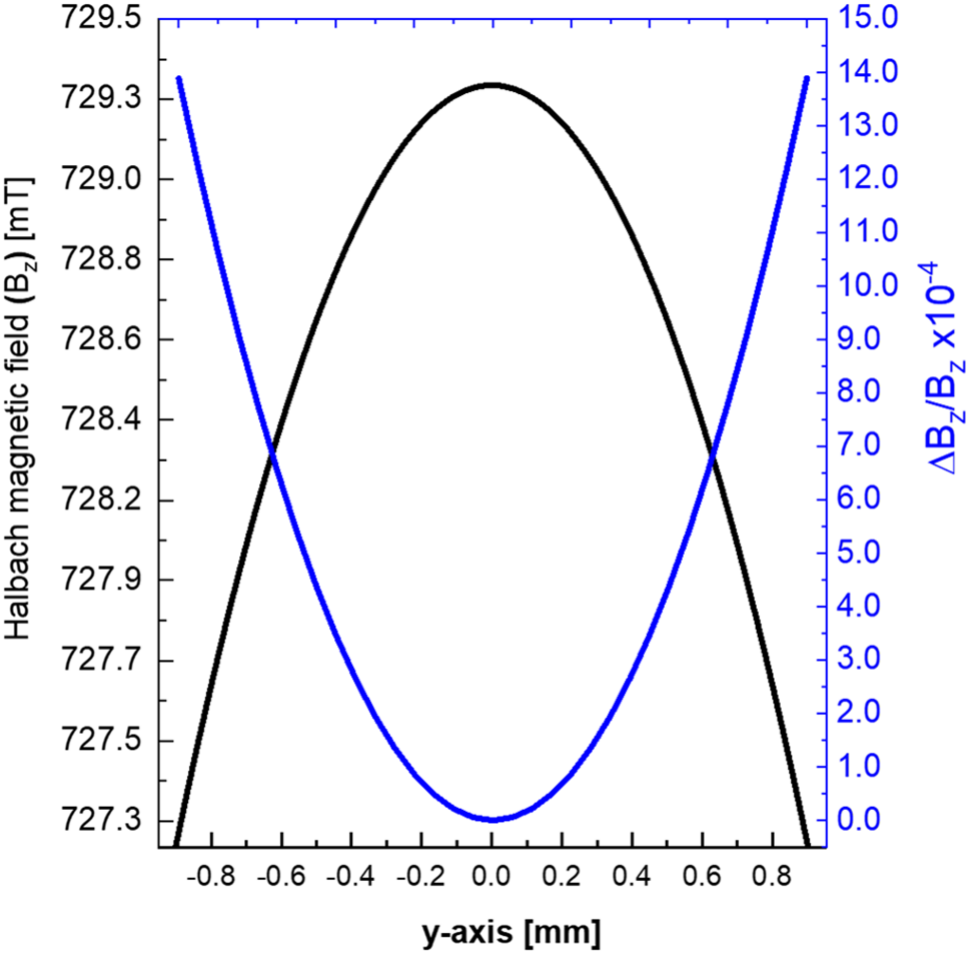
The absolute magnetic field along the x-axis (black curve) for a 3-concentric-rings Halbach magnet. This magnet configuration results in the summation of the z component of the magnetic field to form ${B}_{z}$, while the magnetic field components in the x and y directions become negligible at the sweet spot. The blue curve represents the relative field inhomogeneity, $\Delta {B}_{z}/{B}_{z}$.

Figure 2 illustrates the numerical solution of Eq. (5) for the field along the x-axis. This numerical solution is derived for the finite stack, which is oriented in the x-direction, of the multilayer Halbach magnet comprising the three layers.

The relative field inhomogeneity ($\Delta {B}_{z}/{B}_{z}$) along the x-axis was analytically calculated, following the comprehensive description by Soltner and Blümler^24^. Consequently, the Multilayer Halbach Magnet (MLHM) exhibits a field inhomogeneity of approximately 140 ppm across the sample volume in the absence of the combined shimming system. Specific parameters, including, ${r}_{inner}$,${r}_{outer}$, and $r$ for the completed Halbach magnet, are provided in Table 1.

**Table 1:** The key parameters for the 3-Halbach layers and the rotatable shimming layer, including ${r}_{inner}$,${r}_{outer}$ and $r$ values.

$Layer$(*l*) [#]	$n [\#]$	${r}_{inner} [\mathrm{mm}]$		${r}_{outer} [\mathrm{mm}]$		$r [\mathrm{mm}]$		$Size [\mathrm{mm}]$	${B}_{R} [\mathrm{mT}]$	${B}_{z} \left(x\right)at (x,y,z=0)$ $[\mathrm{mT}]$
		Cal.	Mea.	Cal.	Mea.	Cal.	Mea.	As received		Cal.
1	16	15.9	16.0	25.1	25.2	20.5	20.5	$6\times 6$	1350	230.6
2	16	24.8	25.0	37.95	37.75	31.0	31.0	$10\times 10$	1350	215.3
3	12	35.5	36.0	60.0	60.2	47.0	47.0	$20\times 20$	1350	282.9
Mini-ring Halbach mechanical shimming [layer 0]										
0	20	11.2	11.4	18.3	18.7	13.8	14.1	$3\times 3$	1320	123.9

Additionally, it lists the sizes and magnetic remanence (${B}_{R}$) of the cubic magnet elements within each layer, along with the magnetic field ${B}_{z} \left(x\right)$ at the coordinates ($x, y, z = 0$).

Cal.: calculated; Mea.: measured.

## Fabrication and characterization

### Multilayer Halbach magnet

**Figure 3:**
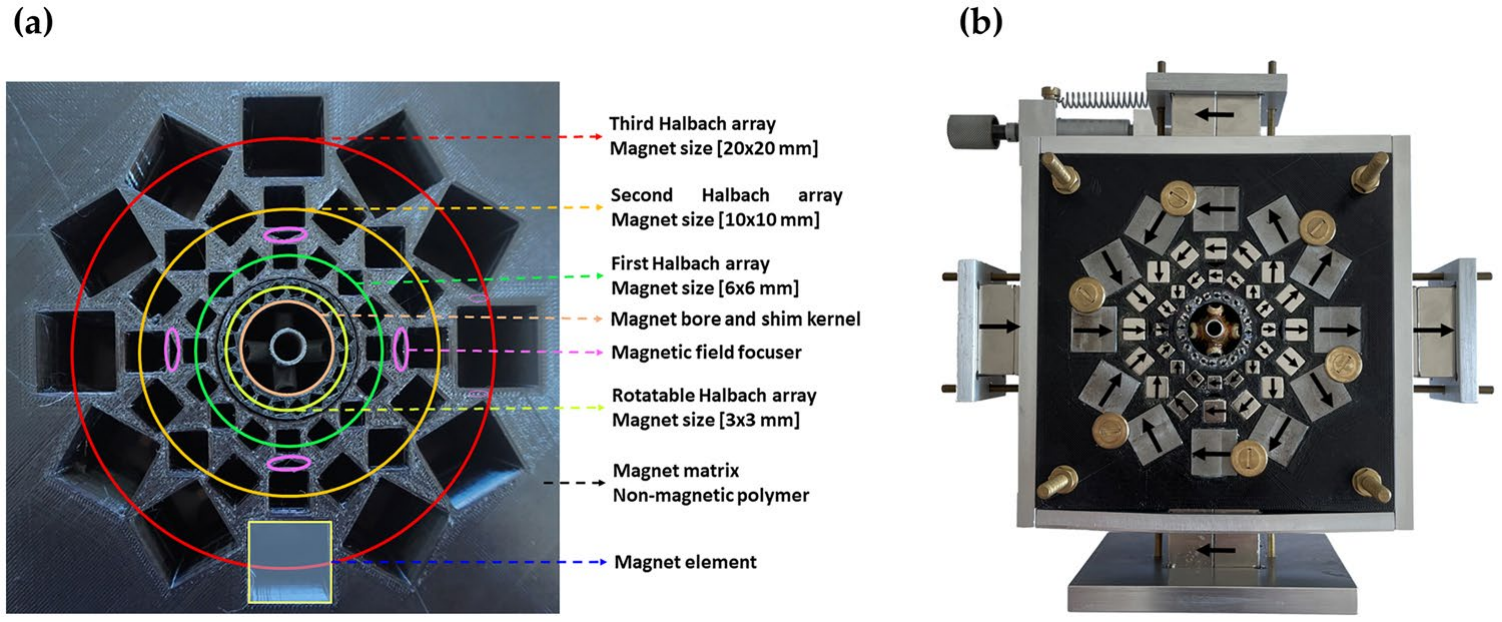
(**a**) Multilayer Halbach magnet designed using 3D CAD and finalized with a 3D printer. Three layers, rotatable components, magnetic focusers, and magnet elements are color-coded for clarity. (**b**) The fully assembled Halbach magnet includes the mechanical and electrical shimming components, along with the shimming kernel.

The Multilayer Halbach Magnet (MLHM) developed in this study is a three-layer cylindrical magnet with a nested, rotatable inner layer (Halbach array) for mechanical shimming (Fig. 3a). The design of the rotatable mechanical shimming layer was carried out using 3D CAD and finalized using a high-resolution 3D printer (FelixPrinter FFF 3D Printer). Sintered magnet elements of Neodymium-Iron-Boron (NdFeB) grade N42 were purchased (Ningbo Permanent Magnetic Materials Ltd.-NGYC, Yinxian Ningbo). The individual magnets are nickel-coated cubes with side dimensions of $20, 10, 6, \; \mathrm{and} \;\;3\; \mathrm{mm}$ $\pm 0.05 \; \mathrm{mm}$. The remanence is ${B}_{R}=1350\; \mathrm{mT}$, coercivity ${H}_{c}=923 \; {\text{kA}}/\text{m}$ and maximum energy product ${(BH)}_{max}=318.3\; \mathrm{KJ}/{\mathrm{m}}^{3}, =42 \; \mathrm{MGOe}$. Furthermore, the completed Halbach array includes a quadrupole layer comprising four ferromagnetic cubes, each with a side dimension of 40 mm, made of ferromagnetic steel and coated with nickel. This quadrupole layer is designed to manipulate magnetic field lines, concentrating and bending them to strengthen the magnetic field and minimize magnetic stray fields^29^ (Fig. 3b). The focusers are simply an approach to design an anti-bend magnet based on a quadrupole^30^. The focusers, in the form of triangular cavities with a 20 mm base and 3 mm height, are oriented as shown in Fig. 3a and filled with iron powder to enhance the magnetic field at the sweet spot. The arrangement of magnetic elements in each layer follows a cubic void alignment based on Eq. (3) and is illustrated in Fig. 3a.

To facilitate mechanical shimming, the completed magnet was enclosed with a 5 mm thick aluminum frame, allowing the mounting of a micrometer screw. The magnetic field strength at the nominal sweet spot within the magnet bore was fine-tuned by inserting an ultrafine polished ferromagnetic cylinder made of ferritic steel. This cylinder serves as a filler to normalize the stray magnetic field lines and reduce the magnetic field strength, enabling operation at lower frequencies^31,32^.

### ${{{B}}}_{0}$ mapping

**Figure 4:**
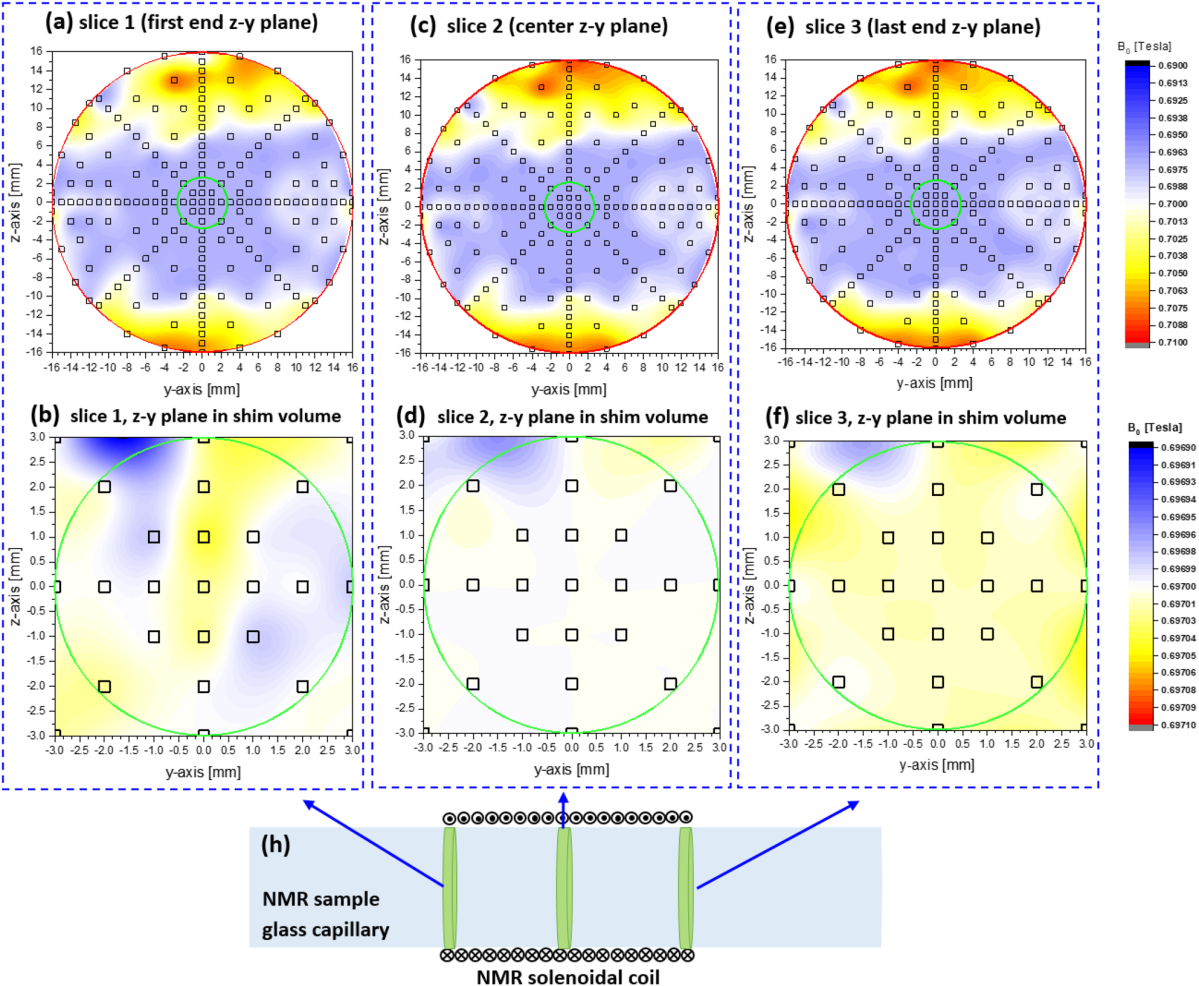
Absolute ${B}_{0}$ mapping was conducted in the 3.4 mm bore of the MLHM magnet without operating the shim system (green circle represents the complete volume). The mapping encompassed three volume slices within the $z{\text{-}}y$ plane, corresponding to the two ends and the center slices (**a**, **b**, and **c**). Subsequently, (**d**, **e**, and **f**) illustrate the absolute ${B}_{0}$ mapping of the usable cylindrical cavity (D = 6 mm, L = 6 mm) (green circles represent the shim-usable volume) after the shim system was inserted. (**h**) Depicts the glass capillary (2.4 mm ID and 3 mm OD) containing the NMR solenoidal coil where the NMR sample is located, showing the three $y{\text{-}}z$ slices within the shim volume.

The absolute ${B}_{0}$ field strength of the MLHM magnet, before equipping it with the shim system, was mapped by placing a directional magnetometer (gaussmeter) inside the magnet bore, aligned parallel to the $z{\text{-}}\mathrm{axis}$. Precise positioning of the magnetometer was achieved using a high-precision 3-axis stage micrometer (Thorlabs, Inc.). The ${B}_{0}$ mapping process encompassed the entire usable volume (D = 6 mm, L = 6 mm) (indicated by the red circles in Fig. 4). Furthermore, ${B}_{0}$ mapping was performed at three specific slices located at − 1.5 mm, 0 mm, and 1.5 mm within the confined volume later occupied by the solenoid coil (marked by green circles). The mapping initiated from the nominal sweet spot of the magnet and systematically moved to cover 250 data points within the $z{\text{-}}y$ plane for each of the volume slices. These measurements were carried out by inserting a magnetometer attached to a 3-axis stage micrometer into the magnet bore. The absolute mapping, conducted prior to inserting the shim system, offers valuable insights into the overall field inhomogeneity of the magnet. This information is crucial for identifying a shim-usable volume, which corresponds to the range of electrical shimming capabilities. The distribution of magnetic field strengths, calculated from the measured data points, is represented in the density plot shown in Fig. 4a–f. These plotted points cover the $z{\text{-}}y$ plane at $x = -1.5$ mm, 0 mm, and 1.5 mm.

The shim process aimed to rectify the magnetic field inhomogeneities within the available cylindrical cavity of the MLHM magnet ($\mathrm{D}=6$ mm, $\mathrm{L}=6$ mm, as denoted by the inner green circles in Fig. 4) by employing a combined mechanical and electrical approach (refer to “Mechanical shim system” and “Electrical shim system” sections). The effective shim was applied to the volume fraction occupied by the sample within the solenoidal coil, which was approximately 3 mm in length and had a 3 mm inner diameter (ID). However, the entire cylindrical cavity ($\mathrm{D}=6$ mm, $\mathrm{L}=6$ mm) was shimmable. The probehead's positioning capabilities within the $y{\text{-}}z$ plane had a range of 1.2 mm, which proved entirely adequate for identifying the most homogeneous volume to encompass the sample, thereby achieving the narrowest line width and optimal signal shape.

### Mechanical shim system

**Figure 5:**
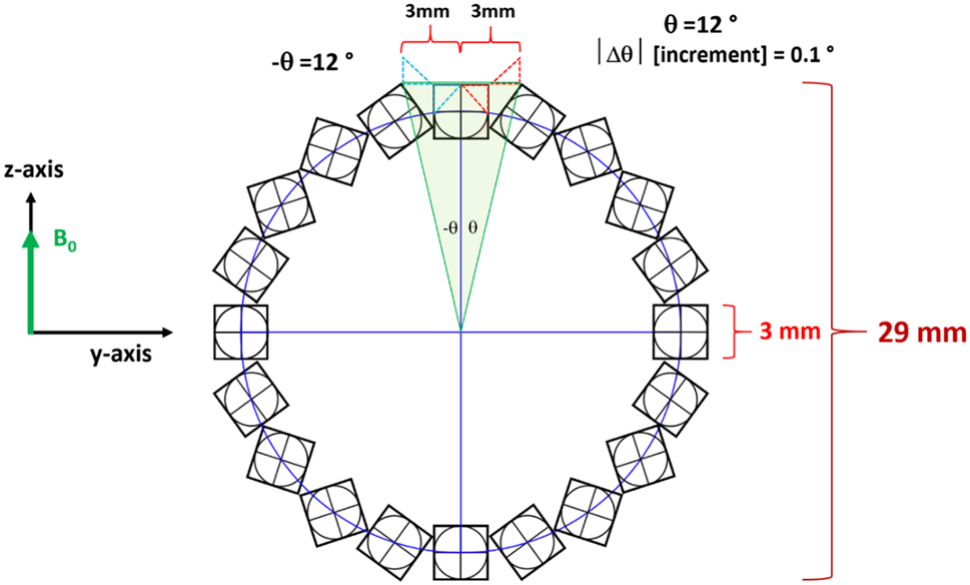
The rotatable Halbach layer served as an integrated mechanical shim system for adjusting ${B}_{z(z{\text{-}}y \;plane)}$ through a limited rotation within the $z{\text{-}}y$ plane ranging from $-12^\circ$ to $+12^\circ$.

A rotatable homemade magnetic layer, configured in the Halbach form (Fig. 5), was installed as the initial layer of the MLHM magnet (highlighted in yellowish-green and labeled in Fig. 3) to change the orientation of the magnetic field vector within the $z{\text{-}}y$ plane.

The rotatable shim layer is secured to a custom-made spring-loaded micrometer, enabling a reversible and precise rotational adjustment of 1⁄120 of the cubic element's dimension within the shim ring (3 mm). The mechanical shimming system contributes to the magnetic components of ${B}_{z(mechanical)}$ and enhances the ${B}_{z}$-component of ${B}_{0}$. To achieve greater ${B}_{z}$ homogeneity, fine shimming is necessary in $\mathrm{X},\mathrm{ Y},\mathrm{ Z},\mathrm{ ZX},\mathrm{ ZY},\mathrm{ XY \;\; and } \; \;{\mathrm{Z}}^{2}$ channels. Therefore, we incorporated an electrical shim system to meet these requirements.

### Electrical shim system

**Figure 6:**
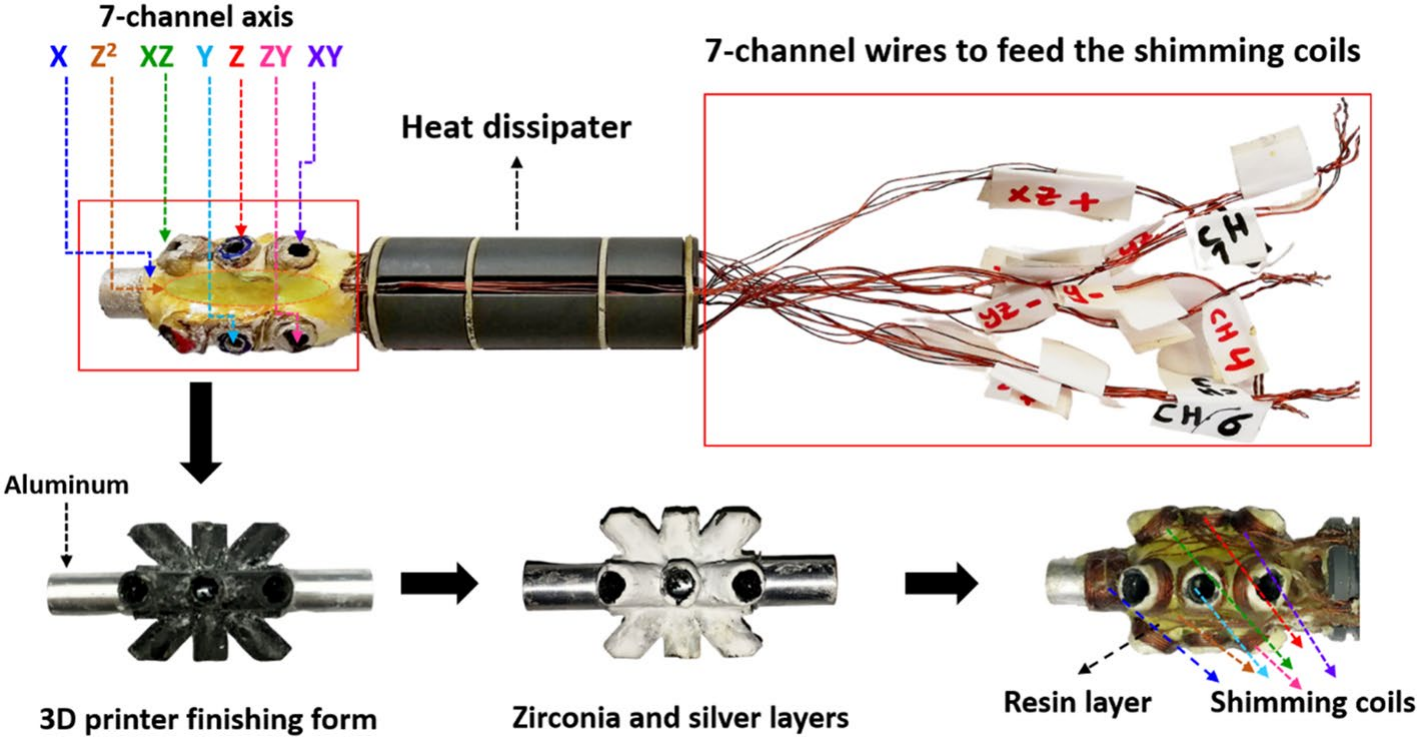
The shimming kernel featuring 7-Helmholtz axis coils, designed using 3D CAD and produced through 3D printing. Each axis's coils are divided into 4 parallel paths to minimize ohmic heating.

The wide-bore magnet was designed to accommodate the electrical shimming system, as shown in Fig. 6. This 7-channel shim system includes a shim kernel in a Helmholtz configuration, which supports the standard gradient shim coil design. The electrical component of the shim kernel is connected to a programmable homebuilt digital 7-channel current source, capable of providing up to $\pm 2 \;\mathrm{ A}$ per channel with a resolution of 0.05 A. To fit within the narrow bore of the Halbach magnet used in this work, some shimming channels and higher orders were omitted. Instead, we replaced the typically used flexible printed circuit board (PCB, FR-4) with copper shim coils wound on hollow tubes, integrated as part of the shim kernel, as depicted in Fig. 6. The design of the shim kernel was created using 3D CAD and was finalized by employing a 3D printer (Dremel DigiLab 3D45 3D Printer). In the original shim system, the addressed shim coils included X, Y, Z, XY, XZ, YZ, X^2^ − Y^2^, and 2Z^2^ − X^2^ − Y^2^ shim coils. However, in this work, we focused on Z^2^, X, Y, Z, XY, XZ and YZ shims based on the 3D configuration. This choice was made for (i) These axes are the most influential in achieving a uniform magnetic field at the sweet spot. (ii) The available space within the extremely small cavity needed to accommodate the sample, shim kernels, wires, and passive heat dissipation. (iii) Higher-order harmonic shims would have required lithographic patterns on a PCB, which could not be mounted within the shim kernel. Therefore, we selected the higher order of Z^2^, which could be managed with 5 copper wires in an elliptical form connecting all channels, forming the second order as described in the literature^33^. For other channels, we established a reference with the X shim coil axis. Subsequently, the X shim coil, which rotates 90° around the z-axis, was designated as the Y shim coil, with the z-axis fixed in the direction of the Halbach magnet's ${B}_{0}$. The XZ shim coil rotates by 45° around the Y-axis, and correspondingly, the XZ shim coil that rotates 45° around the z-axis was labeled as the YZ shim coil^34,35^. The copper shimming coils were coated with three layers. First, a layer of zirconia, an excellent electrical insulator with high thermal conductivity (thermal conductivity of 8.1 W/m K^36^) was applied to provide both electrical insulation and mechanical protection for the coils. Second, an epoxy layer was added on top of the zirconia due to its high heat conduction and low heat capacity. Finally, a silver-based resin was applied to enhance thermal conductivity, facilitating greater passive heat dissipation. These three layers were coated with thicknesses ranging from 200 to 500 μm to prevent thermal storage that could lead to temperature overshoot.

**Figure 7:**
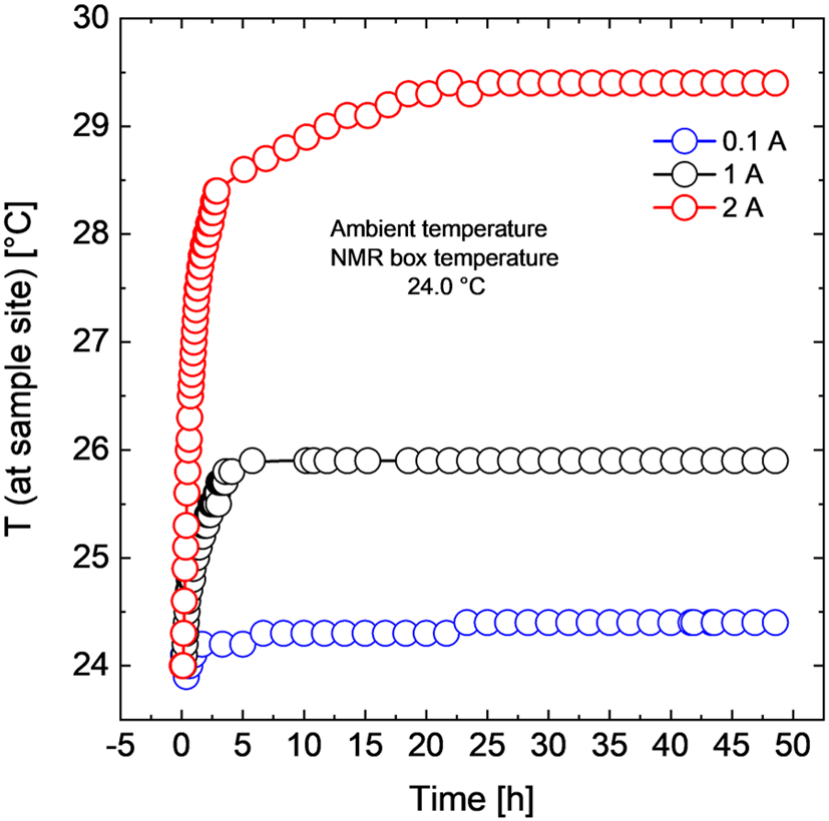
Temperature changes at the sample site during operation of the electrical shim system at currents of 0.1 A, 1.0 A, and 2.0 A per shim channel, illustrating the effectiveness of heat dissipation from the sample site.

Passive heat dissipation and transfer using the shim kernel were adopted in this study, including its extension beyond the magnet cavity. Power dissipation is directly related to the total mass of the dissipater. The shim kernel has a volume of approximately 55 55 cm^3^ and a mass of approximately 18 g, with an effective surface area, including the internal components, of 105 cm^2^. To address the Joule heating generated by the shim system, especially when operating the shimming current at maximum levels ($\pm 2 \; \mathrm{ A}$ for each channel), each shim coil per channel was split into 4 coils connected in parallel to reduce electrical resistance. Consequently, the running electrical currents were reduced to a quarter of the main channel current, resulting in a fourfold reduction in dissipated heat (${\mathrm{E}}_{dis}={\mathrm{I}}^{2}\mathrm{R}$, where $\mathrm{I}=$ electrical current, and $\mathrm{R}=$ Ohmic resistance) at the sample spot. This reduction led to a decrease in temperature from 96.2 to 32.6 °C at full operation currents (14 A for all channels). While thick copper wires can reduce Joule heating, it's important to note that the magnetic field strength of a coil is related to the ampere-turns of the coil. More turns result in a greater induced magnetic field strength. Thick copper wires may limit the ability to fulfill this condition, especially in the limited length (approximately 5 mm) of the stages within the shim kernel. In conclusion, by implementing the two modifications, the electrical shim system was able to operate at typical shim currents of around 0.1 A per channel without causing a significant increase in sample temperature (approximately $0.1 \; ^\circ \mathrm{C}$), as illustrated in Fig. 7 (blue data points). While operating the 7-axes shimming coils at maximum current is rare, testing under extreme conditions involved operating all shim channels at half of the maximum and full maximum currents (1 A and $2\;\mathrm{ A}$, respectively) for validation purposes. Under these conditions, the shim-kernel system exhibited stability, with temperature increases above room temperature measured at $0.3\;^\circ \mathrm{C}$, $3.0\;^\circ \mathrm{C}$, and $5.0\;^\circ \mathrm{C}$ for currents of $0.1$ $\mathrm{A}$, $1\mathrm{ A}$, and $2\mathrm{ A}$, respectively.

### Planar NMR probehead

**Figure 8:**
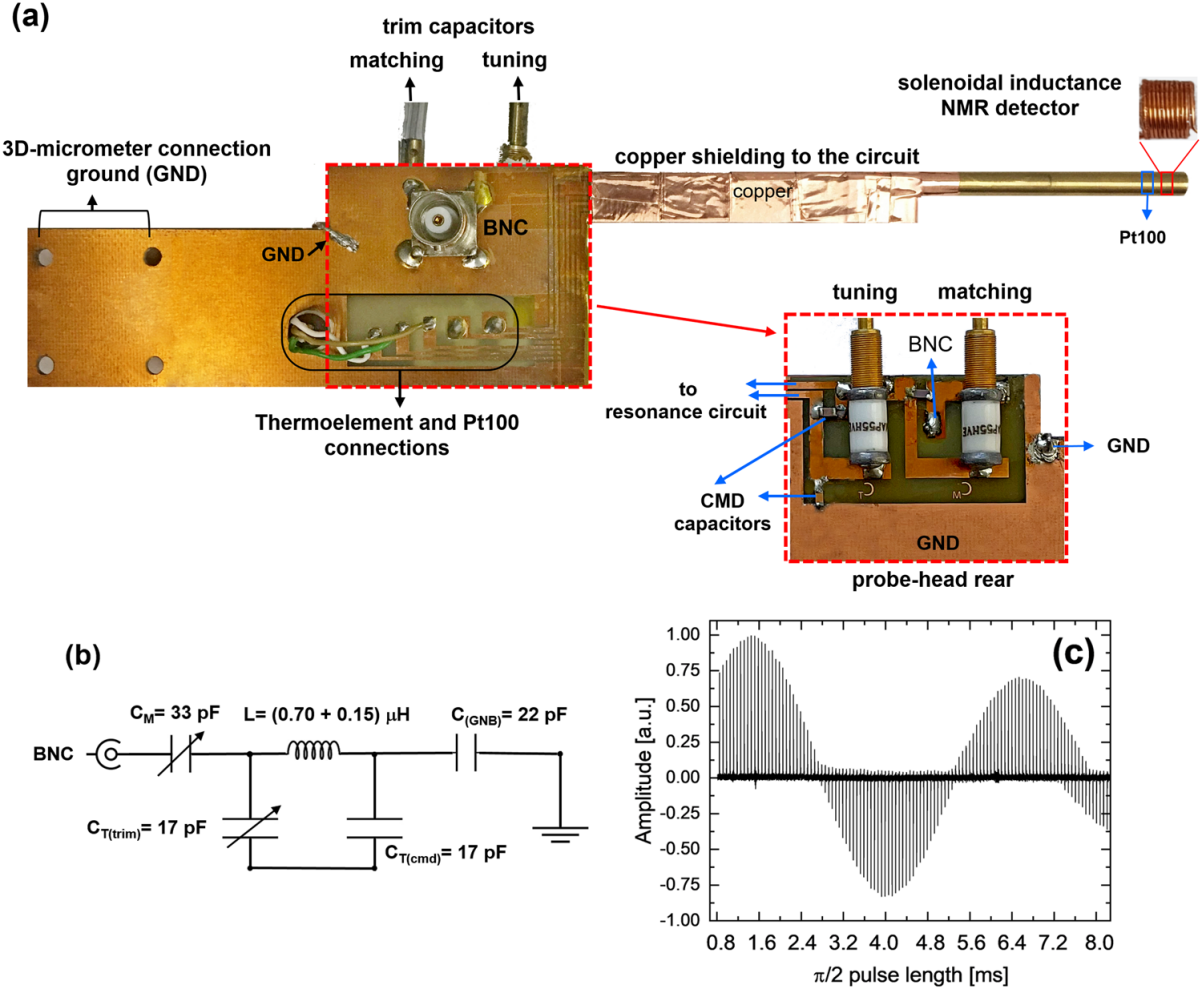
(**a**) Solenoidal-based NMR resonator (probehead) including on-chip thermal elements, a BNC r.f. connector, and electrical connections. (**b**) The electrical circuit of the NMR probehead tuned and matched at 29.934 MHz, showing the tuning and matching capacitors and the ohmic resistance of the circuit. The coil consists of 11 turns, with a 3.4 mm OD, 3 mm ID, and an inductance of approximately 0.70 µH, using a copper wire with a diameter (D) of 0.2 mm. The inductance of the onboard copper strip line connectors is approximately 0.15 µH. **(c)** The nutation curve obtained with the probehead in (**a**).

An NMR probe (Fig. 8a) was constructed on a printed circuit board made of glass-reinforced epoxy laminate (RT4) with a double copper layer, each with a thickness of 35 µm^37^. The layout of the resonance circuit and the r.f. BNC copper tracks were created on the NMR probehead board using UV lithography. The NMR resonator is a solenoidal coil with a copper wire diameter (D) of 200 µm, consisting of 11 turns wound around a high-resolution NMR glass tube with an outer diameter (OD) of 3 mm and an inner diameter (ID) of 2.4 mm. The solenoidal coil accounts for the major part of the inductance (approximately 0.70 μH), while the onboard copper track connectors contribute about 0.15 μH to the resonance circuit (Fig. 8b). Bonding wires and connection points introduce impedance discontinuities, leading to increased transmission loss and signal reflection^38^, particularly as resonance frequency increases^39^, even at low-field NMR. Therefore, the NMR probe was designed as a solenoidal mini coil integrated into a planar design to enhance sensitivity, minimize B1 inhomogeneity, and reduce the impact of bonding wires and connection points on impedance discontinuity and signal transmission loss. Nonmagnetic trim multi-turn and ceramic chip capacitors (Voltronics Corp., Salisbury, MD, USA) were used to tune and match the resonance circuit to 29.934 MHz. The nutation spectrum (Fig. 8c) for the mounted solenoid mini-coil in the probe-head demonstrates the strength and homogeneity of the r.f. field. In this case, the signal intensity as a function of pulse length reveals a strong r.f. field at a power of only 2 W. Sample temperature was detected using a Pt100 sensor positioned near the sample. Temperature calibration at the sweet spot was achieved based on the chemical shift differences of ethylglycol^40^. The electrical circuit of the NMR probe-head, including matching and tuning capacitors, is shown in Fig. 8b. The probe-head successfully achieved a hard pulse length of 1.55 μs at 2 W.

The sensitivity of the homebuilt planar NMR probe, in the NMR experiment at a magnetic field strength of 0.703 T (29.934 MHz for ^1^H), is determined using the signal-to-noise ratio (SNR), typically defined as the ratio of the height of a resonance of interest to twice the root-mean-square of the noise level. In this study, the SNR of the anomeric proton of sucrose was measured to quantify the sensitivity of the planar NMR probe, following a typical protocol in the literature^41^. The sucrose sample had a volume of approximately 35 µL at a concentration of 21.8 mM in ${\mathrm{D}}_{2}\mathrm{O}$, but the SNR is directly proportional to the volume fraction occupied by the sample within the coil, making the effective sample volume around 14 µL. With an experiment time of 8 accumulation scans, each taking 1.9 s per scan, and an SNR of 116, the limit of detection (${n\mathrm{LOD}}_{m}$) is calculated using the formula ${n\mathrm{LOD}}_{m}=79 \; \text{nmols}^{1/2}$ at 29.934 MHz calculated from the formula ${n\mathrm{LOD}}_{m}=3n\sqrt{{t}_{exp}}/\mathrm{SNR \;\;where} \;\; n$ [mol] refers to the portion of the sample within the 14 µL detection volume, and ${t}_{exp}$ is the total experiment time (accumulated over 8 scans). To compare this measurement to one conducted at an NMR frequency of 600.13 MHz, the result was normalized by multiplying it by a factor of 189, following the scaling relationship ${\left[{\upomega }_{L}\right]}^{7/4}$^42,43^. The LOD falls within the range of 15–30 mg/L.

**Figure 9:**
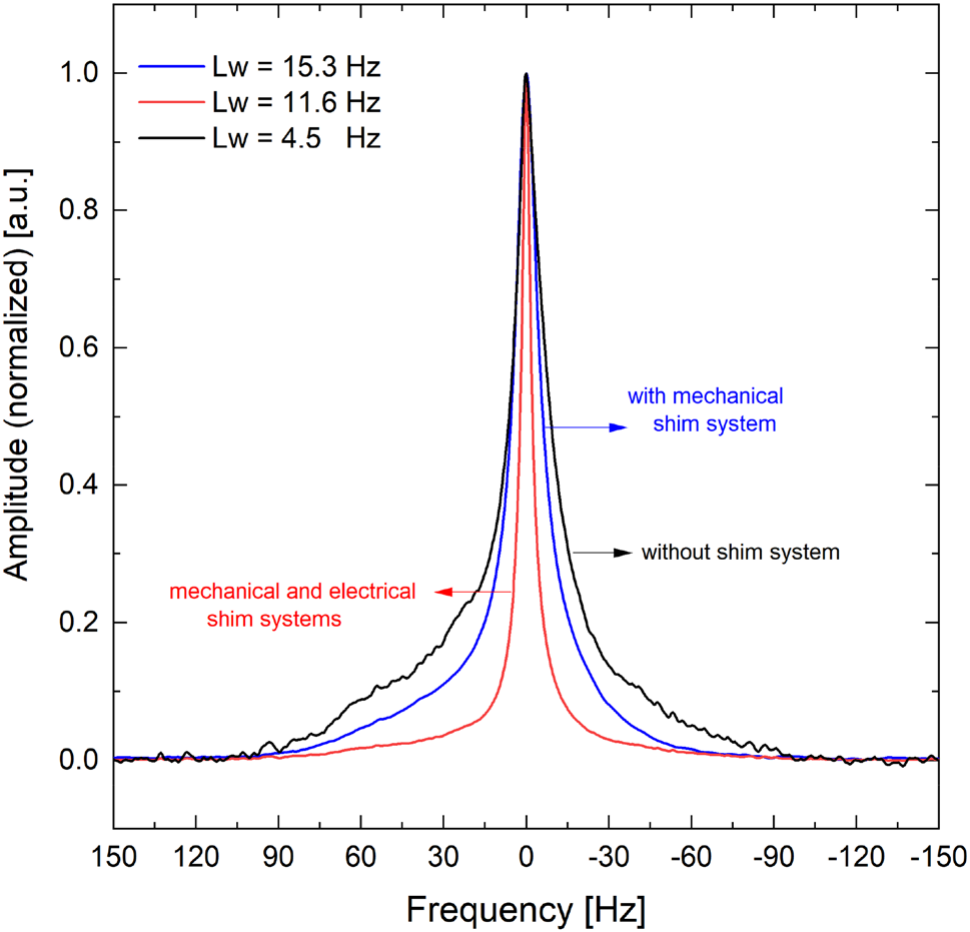
Water signals acquired without a shimming system (black), with mechanical shimming (blue), and with combined mechanical and electrical shimming (red).

Water signals were obtained using the onboard NMR probe within a 3.0 mm OD and 2.4 mm ID NMR glass tube, with a length of 3 mm, as shown in Fig. 9. The black spectrum represents data acquired without any shimming system, resulting in a linewidth of 11 Hz and a non-pure Lorentzian spectral line shape due to the influence of ${\mathrm{B}}_{0}$ magnetic field inhomogeneity. In contrast, the blue spectrum, acquired with mechanical shimming alone (no electrical shimming), demonstrates noticeable improvements, with a reduced linewidth of approximately 8.0 Hz and a mostly Lorentzian shape. The red spectrum, acquired with both mechanical and electrical shimming, exhibits a further reduction in linewidth to 5.5 Hz and a purely Lorentzian signal shape, indicating significantly enhanced homogeneity of the ${\mathrm{B}}_{0}$ magnetic field.

### Low-field NMR spectrometer

**Figure 10:**
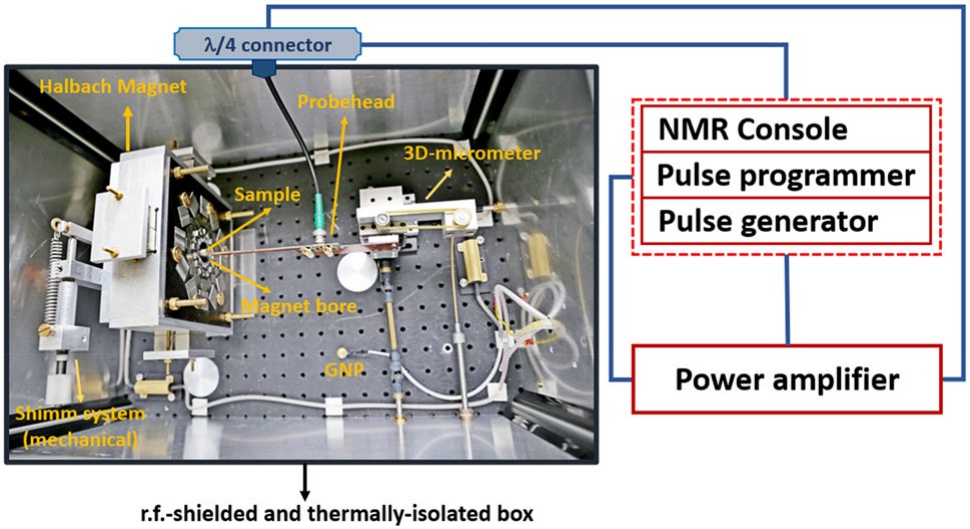
The homebuilt LF-NMR system equipped with an MLHB magnet, labeled with its components: NMR probe-head, ground coupling (GND), multilayer Halbach magnet (MLHB), mechanical shim system, 3D-stage micrometer, thermally isolated and r.f.-shielded box, NMR console (pulse programmer and pulse generator), and power amplifier.

The homebuilt LF-NMR spectrometer (Fig. 10) is equipped with an MLHB magnet operating at 29.934 MHz and is connected to the Pulse Blaster interpreter (SpinCore Technologies, Inc., Gainesville, FL, USA) for instrument control. The magnetic field strength of the MLHB magnet can be adjusted within the range of 0.5–0.77 T based on the thickness of the ferromagnetic cylindrical filler in the magnet's cavity (as explained in “Multilayer Halbach magnet” section). Consequently, the spectrometer can operate at frequencies ranging from 21.25 to 32.78 MHz for protons, requiring appropriate tuning and matching of the NMR probehead. The Pulse Blaster interpreter (SpinCore Technologies, Inc., Gainesville, FL, USA) was utilized for instrument control.

The magnet used exhibits a ${\mathrm{B}}_{0}$ homogeneity of ${\Delta \mathrm{B}}_{0}/{\mathrm{B}}_{0}={10}^{-4}$ within a cylindrical free volume measuring 2.4 mm in diameter and 3 mm in length (coil length), which is larger than the intended sample size to prevent linewidth broadening. The NMR probe-head was mounted on a high-precision 3-axis stage micrometer to locate the magnet's sweet spot for conducting NMR measurements. Additionally, a high-precision rotation stage, adjustable in 1° increments (Thorlabs Inc.), was employed to rotate the entire magnet relative to the probe-head, enhancing the alignment (${\mathrm{B}}_{0}$ perpendicular to ${\mathrm{B}}_{1}$) between the magnet and the probehead. This alignment improvement contributed experimentally to achieving narrower linewidth. The temperature control system for both the magnet and the NMR probe was housed in a homebuilt r.f. shielded box made of 2 mm thick aluminum. The box was thermally insulated using a formfitting polyurethane foam jacket and equipped with a heating system. Temperature regulation was achieved through a standalone programmable commercial temperature controller, and passive vibration isolation measures were implemented to suppress mechanical vibrations.

**Figure 11:**
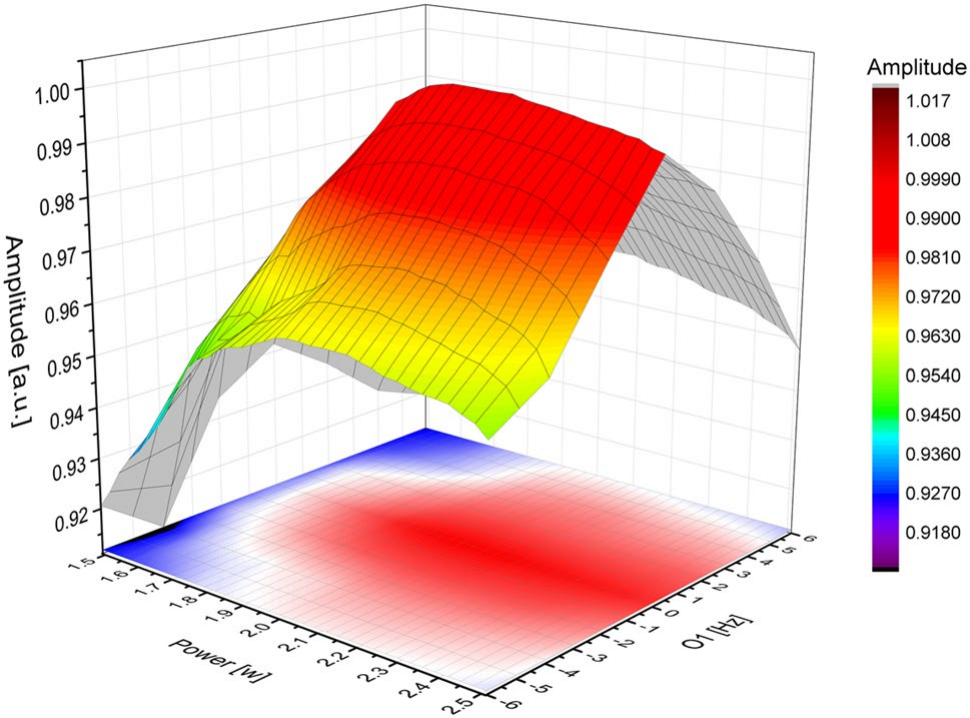
A 3D plot illustrating the variations in NMR signal amplitude as a function of B_1_ power (power of the 90° hard pulse) and central resonance frequency (O1) of ${\mathrm{B}}_{0}$.

Amplitude-modulated pulses were generated using a Radio Processor board (SpinCore Technologies, Inc., Gainesville, FL, USA). Figure 11 presents a 3D plot illustrating the measured NMR signal amplitude variations in response to changes in the power of 90° pulses (with a fixed pulse duration optimized separately at 1.9 ms) and the central resonance frequency (O1) of ${\mathrm{B}}_{0}$. The analysis aimed to determine the parameters for effective and optimized NMR measurements, revealing that a power range of 1.9–2.5 W and a central resonance frequency within ± 5 Hz yielded optimal results, with only a 7% decrease in amplitude even at off-resonance frequencies within this range.

## Discussion and applications

### Selective excitation pulses

Spin system editing is achieved using the Krotov algorithm from optimal control theory, which employs a gradient-based approach to monotonically enhance the objective function at each iteration^44^ and incorporates second-order derivatives to improve convergence, particularly near the optimum^45^. Further details about the Krotov algorithm can be found in^46^. Neat acetic acid, selected due to its non-coupled system nature and a difference of approximately 9.8 ppm between the two targeted NMR signals (hydroxyl and methyl protons signals), serves as the test subject for assessing the Krotov algorithm's performance in low-field NMR.

The Hamiltonian for neat acetic acid is described as follows:

6 $${\mathrm{H}}_{1}=2\uppi ({{\upnu }_{1}^{\mathrm{a}}\mathrm{A}}_{\mathrm{z}}+ {\upnu }_{1}^{\mathrm{b}}{\mathrm{B}}_{\mathrm{z}}+ {{\upnu }_{1}^{\mathrm{c}}\mathrm{C}}_{\mathrm{z}}+ {{\upnu }_{1}^{\mathrm{d}}\mathrm{D}}_{\mathrm{z}})$$

where *a, b* and* c* represent the spins of the methyl group, while d represents the spin of the hydroxyl group of acetic acid. The spin matrices are denoted as A_i_ to D_i_ with $\mathrm{i}$ corresponding to the x, y and z. The resonance frequencies for methyl group are ${\upnu }_{1 }^{\mathrm{a}}=$ ${\upnu }_{1}^{\mathrm{b}}={\upnu }_{1}^{\mathrm{c}}=-140.8\;\mathrm{ Hz}$, and for hydroxyl group it is ${\upnu }_{1}^{\mathrm{d}}=140.2\;\mathrm{ Hz}$.

The calculations were performed as state-to-state transfers, with the initial and terminal operators defined for both systems as follows:

#### Spin Group 1 (methyl group excitation)

7 $$\mathrm{Initial \;operator}\; 1{:}\,\,\,\,\,\,\,\,\,\,\,\,\,\;{\mathrm{I}}_{\mathrm{ini}}^{(1)}={\mathrm{A}}_{\mathrm{z}}+ {\mathrm{B}}_{\mathrm{z}}+ {\mathrm{C}}_{\mathrm{z}}$$

8 $$\mathrm{Initial \;operator} \; 2{:}\,\,\,\,\,\,\,\,\,\,\,\,\,{\mathrm{I}}_{\mathrm{ini}}^{(2)}={\mathrm{D}}_{\mathrm{z}}$$

9 $$\mathrm{Terminal \;operator}\;1{:}\,\,\,\,\,\,\,\,\,\,\,\,\,{\mathrm{TO}}_{1}={\mathrm{A}}_{\mathrm{x}}+ {\mathrm{B}}_{\mathrm{x}}+ {\mathrm{C}}_{\mathrm{x}}$$

10 $$\mathrm{Terminal \; operator} \; 2{:}\,\,\,\,\,\,\,\,\,\,\,\,\,{\mathrm{TO}}_{2}={\mathrm{D}}_{\mathrm{z}}$$

#### Spin Group 2 (hydroxyl group)

11 $$\mathrm{Initial \;operator}\;1{:}\,\,\,\,\,\,\,\,\,\,\,\,\,{\mathrm{I}}_{\mathrm{ini}}^{(1)}={\mathrm{A}}_{\mathrm{z}}+ {\mathrm{B}}_{\mathrm{z}}+ {\mathrm{C}}_{\mathrm{z}}$$

12 $$\mathrm{Initial \;operator}\;2{:}\,\,\,\,\,\,\,\,\,\,\,\,\,{\mathrm{I}}_{\mathrm{ini}}^{(2)}={\mathrm{D}}_{\mathrm{z}}$$

13 $$\mathrm{Terminal \;operator}\;1{:}\,\,\,\,\,\,\,\,\,\,\,\,\,{\mathrm{TO}}_{1}={\mathrm{A}}_{\mathrm{z}}+ {\mathrm{B}}_{\mathrm{z}}+ {\mathrm{C}}_{\mathrm{z}}$$

14 $$\mathrm{Terminal \;operator} \; 2{:}\,\,\,\,\,\,\,\,\,\,\,\,\,{\mathrm{TO}}_{2}={\mathrm{D}}_{\mathrm{x}}$$

The fidelity of the calculation (U_1_ and U_2_ represent the calculated pulse propagators for spin systems 1 and 2, respectively) is determined by:

15 $$fidelity = real\,(Trace({\text{TO}}_{1}^{\prime} {\text{U}}_{1} {\text{I}}_{ini}^{(1)} {\text{U}}_{1}^{\prime} )) + real\,(Trace({\text{TO}}_{2}^{\prime} {\text{U}}_{2} {\text{I}}_{ini}^{(2)} {\text{U}}_{2}^{\prime} ))$$

The pulse calculations were performed using MATLAB version R2019a, utilizing an algorithm based on code from the literature^44^. The optimal control pulses had a duration of 50 ms, and typically, 1000 iterations of the algorithm were employed to achieve convergence. This resulted in calculation times of a few minutes when executed on an INTEL Core i7 system with 8 GB RAM. Experimental ^1^H-NMR spectra were acquired by applying hard pulse and selective excitation pulses using LF-NMR at approximately 0.7 T.

**Figure 12:**
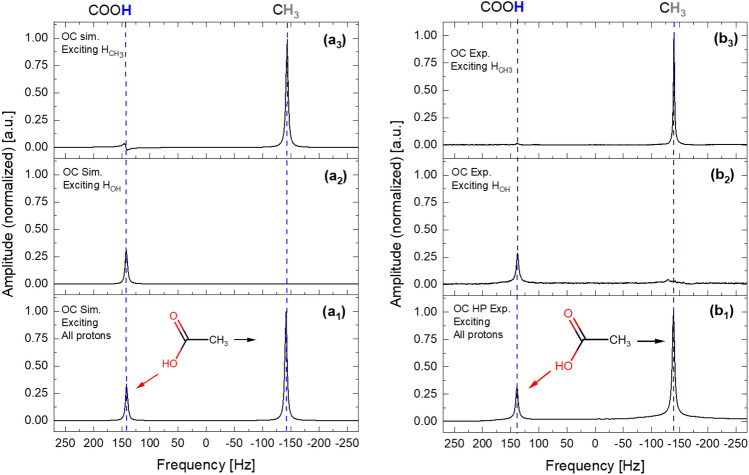
Simulated and experimental ^1^H-NMR spectra at 29.934 MHz of neat acetic acid using optimal control (OC) pulses to excite (**a**_**1**_ and **b**_**1**_) all protons, (**a**_**2**_ and **b**_**2**_) hydroxyl proton, and (**a**_**3**_ and **b**_**3**_) methyl protons. The OC pulses were calculated using amplitude and phase modulation based on the Krotov algorithm.

The corresponding simulated and experimental spectra are displayed in Fig. 12a1, a2, and a3 and b1, b2, and b3, respectively. There is some crosstalk signal from the COOH proton in the simulation, as indicated by Fig. 12a3, and a slight phase error of the methyl signal. Despite acetic acid having no dipolar coupling, Gaussian selective excitation can be used for spectral editing. Nevertheless, this is an excellent example since the two signals appeared experimentally at 2.05 ppm (methyl) and 11.67 ppm (hydroxyl), which are separated by approximately 9.62 ppm (about 285 Hz at an NMR frequency of 29.934 MHz) (Fig. 12b1). The NMR signal of the hydroxyl group in acetic acid shifts toward the average chemical shift between the hydroxyl group in neat acetic acid (11.8 ppm) and the water signal chemical shift (4.75 ppm), considering the concentration of protons on each side^47^. The acetic acid example in this work clearly demonstrates the excitation and suppression efficiency of wanted and unwanted signals without interferences at low-field NMR. Typically, OC pulses for coupling and non-coupling systems are calculated using amplitude and phase modulations. However, for a non-coupling system like acetic acid, exclusively calculated OC based on amplitude modulation is sufficient and has the advantage of avoiding first-order phase errors, making convergence much faster.

Both simulation and experimental spectra demonstrate complete suppression of unwanted signals. Furthermore, the relative amplitudes of the desired signals with respect to the OC-HP remain consistent, showcasing the robust implementation of the optimal control algorithm at the low Larmor frequency. The performance and efficiency of Krotov optimal control pulses compared to Gaussian pulses have been assessed for the hydroxyl and methyl proton groups in neat acetic acid. The applied Gaussian pulse had a 1% cutoff, was offset to the targeted signal, had a maximum power level of 4.25 µW, and a pulse length of 20.4 $\mathrm{ms}$. Excitation and suppression factors (EF and SF, respectively) for both Krotov and Gaussian cases were calculated based on the absolute integration of hydroxyl and methyl proton signals relative to the absolute integration obtained after applying hard pulses, as shown in Table 2. The Gaussian pulse suppressed the unwanted signal with an SF factor of 0.03, similar to the performance of a Krotov selective pulse (SF = 0.04). However, the EF values for the desired signals using a Gaussian pulse (averaging 0.63) exhibit significant differences compared to those achieved with OC pulses (averaging 0.81). Furthermore, Gaussian pulses can only be applied over a continuous range of frequencies, while Krotov selective pulses can target any signal^46^. Notably, the EF is less than 1 since OC pulses operate at much lower power (0.08–0.1 W) compared to hard pulses operating in the power range of (1.9–2.2 W).

**Table 2:** EF and SF factors for signal groups excited with Gaussian and OC (Krotov) pulses.

Factor	Hydroxyl proton OH group		Methyl protons CH_3_ group	
	Krotov	Gaussian	Krotov	Gaussian
EF	0.80	0.62	0.82	0.64
SF	0.04	0.03	0.06	0.05

EF and SF factors are calculated based on the absolute integration of the desired and undesired signals, respectively, in comparison to the absolute integration of these signals after excitation with a hard pulse.

This study has demonstrated the feasibility of applying optimal control pulses for subspectral editing at a field strength as low as 0.7 T. This allows for the use of selective excitation pulses targeting individual components in multicomponent mixtures within the realm of LF-NMR. Such capabilities are of significant interest for selective excitation in applications related to pharmaceutical product characterization and point-of-care diagnostics and applications.

## Conclusions

The applicability of the LF-NMR system across various fields requires innovative developments, particularly in enhancing magnetic homogeneity, sensitivity, and resolution. The integration of a multilayer Halbach magnet, combined mechanical and electrical shimming system, onboard NMR probehead, and selective excitation pulses, as demonstrated in this study, has the potential to accelerate the advancement of portable and low-field NMR. The multilayer Halbach magnet was designed and finished to attain a strong and uniform magnetic field in the main direction (${{\mathrm{B}}_{0}}_{\mathrm{z}}$). The rotatable Halbach array was designed to offer magnetic field shimming in the z-y plane, and it successfully improved homogeneity compared to the standard dipolar permanent magnet^46^. A probehead was manufactured to fit on a single board with minimal electrical jointing points between the electrical components, minimizing impedance discontinuity, transmission loss, and signal reflection. Optimal control pulses based on the Krotov algorithm were successfully implemented for molecular spectral excitation and sub-spectral excitations, even through amplitude modulation, as demonstrated by the spectral editing of neat acetic acid, supporting LF-NMR spectrometer applications, even with limited pulse programmer capabilities. The obtained results show great promise and can be further applied to the targeted analysis of overlapped NMR spectral lines at low magnetic field strengths. This development opens opportunities for using a benchtop NMR spectrometer as an efficient analytical tool, creating significant added value in process analytics.

## Data Availability

The datasets used and/or analysed during the current study available from the corresponding author on reasonable request.
